# Preparation and Microscopic Mechanical Characterization of L-Methionine-Based Polyphosphazene Fibrous Mats for Vascular Tissue Engineering

**DOI:** 10.3390/pharmaceutics15112546

**Published:** 2023-10-28

**Authors:** Meng Wang, Kibret Mequanint

**Affiliations:** Department of Chemical & Biochemical Engineering, The University of Western Ontario, 1151 Richmond Street, London, ON N6A 5B9, Canada; mwang529@uwo.ca

**Keywords:** poly (organophosphazenes), human mesenchymal stem cell(h-MSCs), vascular smooth muscle cells, vascular tissue engineering, atomic force microscopy (AFM), tissue scaffolds

## Abstract

This study investigates the mechanical properties, degradation behavior, and biocompatibility of poly[(α-amino acid ester) phosphazene] electrospun fibers based on the ethyl ester of L-methionine (PαAPz-M), a material with potential applications in tissue engineering. We utilized atomic force microscopy (AFM) to evaluate the fiber mechanical characteristics and calculate its Young’s modulus, revealing it to closely mimic the stiffness of a natural extracellular matrix (ECM). We also studied the degradation behavior of PαAPz-M scaffolds over 21 days, showing that they maintain the highly porous structure required for tissue engineering. Further evaluation of mesenchymal multipotent 10T1/2 cell and mesenchymal stem cell (MSC) behavior on the scaffolds demonstrated significant cell viability, proliferation, and successful MSC differentiation into smooth muscle cells. Expression of collagen and elastin by MSCs on the fiber mats highlighted potential ECM formation during scaffold degradation, confirming PαAPz-M as a promising material for vascular tissue engineering.

## 1. Introduction

Poly[(α-amino acid ester) phosphazene]s, or PαAPz, show promise in the field of tissue engineering and drug delivery due to their unique degradation characteristics. Unlike many biodegradable polyesters, such as poly(lactide) and poly(glycolide), PαAPz release non-acidic and buffering degradation products, primarily comprising phosphate, ammonia, and corresponding side groups [1,2]. Consequently, PαAPz have been reported to mitigate inflammatory responses triggered by acidic degradants when used synergistically with other materials [3,4].

Current applications of PαAPz in tissue engineering have predominantly employed those derived from alanine and phenylalanine, with a principal focus on bone tissue engineering [5,6,7]. This is owed to their glass transition temperatures typically being higher than physiological temperature, ensuring structural integrity and mechanical support in vivo [6,8].

However, vascular tissue engineering scaffolds should be flexible to mimic native blood vessel mechanical properties [9,10]. Therefore, in contrast to bone tissue engineering, vascular tissue engineering may benefit from more pliable materials. Previously, we identified methionine-based polyphosphazene, or PαAPz-M, whose glass transition temperature is approximately 9 °C, as a potential candidate for this purpose [11]. This material is especially appealing considering the sensitivity of vascular smooth muscle cells (VSMCs) to acidic degradation products.

Although synthesis methods for PαAPz-M have been reported [11], a comprehensive study of its influence on cell affinity, differentiation, and the effect of its hydrolysis on cellular adhesion is still lacking.

In this study, we endeavored to elucidate the potential of PαAPz-M for vascular tissue engineering applications. We synthesized and characterized PαAPz-M, fabricated fibrous scaffolds through electrospinning methods, and assessed their performance in vitro. To benchmark our findings, we also conducted a comparative study with poly(ester amide) (PEA), a well-documented synthetic biomaterial used in vascular tissue engineering [12,13].

Given the central role of mechanical behavior in evaluating biomaterials for tissue engineering, we explored the mechanical properties of PαAPz-M [14,15,16]. Macroscopic mechanical properties (e.g., those obtained from tensile testing of fibrous mats) are completely sensed by cells; instead, microscopic and mechanical properties localized at the cellular scale are more important, thus prompting us to use AFM to measure local mechanical properties at the microscale [17,18]. Compared to mechanical tests at the macroscale, measuring the local mechanical properties at the microscale is believed to provide valuable insights into the complex interplay of cell-to-cell and cell-to-material communication [18,19].

To evaluate the degradation rate of PαAPz-M, we performed a 21-day in vitro degradation study of the fiber mat, observing alterations in its surface morphology using SEM. Subsequently, we tested the material’s cell affinity and differentiation potential. For this purpose, we selected two types of cells: a mouse embryonic multipotent mesenchymal progenitor cell line (10T1/2 cells) and human mesenchymal stem cells (MSCs). Here, 10T1/2 cells, known for their easy cultivation and potential to differentiate into VSMCs, served as excellent model cells [20], while MSCs, on the other hand, due to their human source and potential to differentiate into VSMCs, were deemed optimal for human vascular tissue engineering [21,22].

Overall, our research aimed to provide a systematic investigation of a methionine-based polyphosphazene derivative, PαAPz-M, starting from its synthesis and characterization to its scaffold fabrication and evaluation for vascular tissue engineering. We particularly focused on understanding its degradation behavior, mechanical properties, and cell compatibility, and thus its potential as a favorable scaffold for vascular tissue engineering applications.

## 2. Materials and Methods

### 2.1. Materials

Hexachlorocyclotriphosphazene (HCCP) from Sigma Aldrich (Milwaukee, WI, USA) was recrystallized under vacuum at 55 °C. Poly(dichlorophosphazene) (PDCP) was synthesized as reported previously [23]. Anhydrous tetrahydrofuran (THF) and glass distilled hexanes-190 were purchased from Caledon Labs (Georgetown, ON, USA). Triethylamine (Et_3_N), chloroform (CHCl_3_), and dimethyl sulfoxide (DMSO) were purchased from Sigma Aldrich. L-Methionine ethyl ester hydrochloride (H-Met-OEt·HCl) was obtained from Alfa Aesar (Ward Hill, MA, USA) and stored in the fridge. Unless specified otherwise, all chemicals and solvents were used as received.

A polyester amide (PEA), specifically 8-Phe-4, was synthesized by interfacial polymerization using sebacoyl chloride, butanediol, and L-phenylalanine as the amino acid, according to our previous publication [24].

### 2.2. Synthesis of PαAPz-M

To synthesize uncrosslinked PDCP, 2.00 g of purified HCCP was added into a flame-dried ampoule, which was then evacuated under vacuum and sealed using a propane torch. Thermal ring-opening polymerization of HCCP was performed at 230 °C for approximately 72 h, and uncrosslinked PDCP was obtained by three precipitations from THF into hexane, as reported previously [23].

To functionalize the PDCP, a one-step method was employed using H-Met-OEt·HCl. Briefly, 20 mL of the 10 wt.% PDCP in THF, 4 mL of triethylamine and 2.36 g of H-Met-OEt·HCl were added into a flame-dried flask and reacted at room temperature for 72 h. The final product, functional PαAPz-M, was purified by filtering and precipitating from THF into hexane 3 times [25].

### 2.3. PDCP and PαAPz Characterization with ^31^P-NMR and ^1^H-NMR

The polymer structures of PEA and PαAPz-M were evaluated using a Varian INOVA 400 MHz spectrometer (Varian Canada Inc., Mississauga, ON). All chemicals were dissolved in chloroform-D (CDCl_3_) with a concentration of approximately 40 mg/mL for ^31^P-NMR and 5 mg/mL for ^1^H-NMR. Chemical shifts are reported in parts per million (ppm) and referenced to chloroform at δ = 7.27 ppm.

### 2.4. Electrospinning of PαAPz

In this experiment, pure PEA, PαAPz-M, and a 50:50 (by weight) mixture of PEA and PαAPz-M were electrospun. The electrospinning solution formula for PEA was the same as that reported before [26], consisting of CHCl_3_:DMSO = 3:1. The electrospinning solution formula for PαAPz-M was THF:CHCl_3_ = 9:1. The mixture of PEA and PαAPz-M at a ratio of 50:50 was a combination of the two electrospinning solutions. The electrospinning solution was packed into a 0.5 mL glass syringe and connected to a 22 G stainless steel needle. Electrospinning flow rate was controlled by a syringe pump (KD101, KD Scientific, Holliston, MA, USA). The stainless-steel needle was connected to the positive electrode of a low-current power supply (Gamma High Voltage, Ormond Beach, FL, USA) that can provide 7–20 kV, and the rotating collector was connected to the ground to ensure a high potential difference between the electrospinning solution and the collector. Electrospinning was performed at room temperature and 20–25% relative humidity.

### 2.5. Scanning Electron Microscopy (SEM)

The morphology of the fibers and the thickness of the fiber mat were analyzed using a scanning electron microscope (S-3400N, Hitachi, Ltd., Tokyo, Japan). Samples were cut into a 1 × 1 cm^2^ square and then attached to an SEM sample holder using carbon tape. Prior to imaging, samples were coated with 5 nm of osmium using a sputter coating technique (Filgen OPC80T Osmium Plasma Coater, Filgen Nanosciences & Biosciences Inc., Jonoyama, Japan). SEM images were obtained at an acceleration voltage of 5 kV and the diameter of the fibers was analyzed using ImageJ software (version 1.50i). Briefly, images were proessed to enhance the contrast, while the diameter of the fibers was measured by analyzing at least 100 randomly selected fibers in 3 independent SEM figures from each sample. The reported values represent the average of the measurements obtained from at least three independent samples.

### 2.6. Measurement of Mechanical Properties by AFM

Mechanical testing at the microscopic scale was conducted with an atomic force microscope (AFM) (Bruker ICON AFM) using the MultiMode platform. AC 160 silicon nitride cantilevers (Bruker) with a silicon sphere tip with a diameter of 20 μm and a wedge-shaped tip with a diameter of 7 nm were used for AFM mechanical testing. Before testing began, the actual spring constants of AFM cantilevers were measured using the thermal tuning method [27]. Fiber mats with a thickness of 30–70 μm were chosen for mechanical testing. Prior to testing, the fiber mats were cut into pieces with dimensions of approximately 5 mm × 10 mm. The fiber mats were mounted on a glass slide using double-sided adhesive tape, and the AFM cantilever was brought into contact with the sample surface. A force–distance curve was recorded for each sample using the contact mode with a maximum indentation depth of 5 μm and a rate of 1 μm/s. At least 10 force–distance curves were recorded for each sample.

In order to evaluate the mechanical properties of the samples, the Hertz model was employed [28,29]. Typically, the size of the material being tested is much larger than the contact area between the AFM probe and the material. Thus, the Hertz model can be simplified as shown in Equation (1), where *F* is the applied load, *E* is Young’s modulus, ν is Poisson’s ratio of the sample, *R* is the radius of the contact area, and h is the indentation depth [28].

(1) $$F=\frac{4}{3\left(1-\nu^{2}\right)}ER^{\frac{1}{2}}h^{\frac{3}{2}},$$

When fitting a force curve with a Hertz model, there are two different methods of analysis. One is to raise both sides of the equation to the power of 2/3 which results in Equation (2). Since Young’s modulus and Poisson’s ratio are constant, *F*^2/3^ and *h* are linearly related when *R* does not change during the indentation.

(2) $$F^{\frac{2}{3}}={\left[\frac{4}{3\left(1-\nu^{2}\right)}ER^{\frac{1}{2}}\right]}^{\frac{2}{3}}h⟹F^{\frac{2}{3}}=Sh,\mathrm{where}\;S={\left[\frac{4}{3\left(1-\nu^{2}\right)}ER^{\frac{1}{2}}\right]}^{\frac{2}{3}}$$

(3) $$E=\left[\frac{3\left(1-\nu^{2}\right)}{4R^{\frac{1}{2}}}\right]S^{\frac{3}{2}},$$

The other is to use Equation (3) from the Derjaguin–Muller–Toporov (DMT) model and substitute it into Equation (1), resulting in Equation (4). In Equations (3) and (4), *R_p_* represents the diameter of the probe and is constant during the measurement [30,31]. As a result, *F* and *h* behave linearly in the force curve.

(4) $$R_{p}{}^{2}=Rh,$$

(5) $$F=\frac{4}{3\left(1-\nu^{2}\right)}E{\left(Rh\right)}^{\frac{1}{2}}h=\frac{4ER_{p}}{3\left(1-\nu^{2}\right)}h\;\;\;\;\;\;⟹\;\;\;\;\;\;\;F=Sh,$$

(6) $$E=\left[\frac{3\left(1-\nu^{2}\right)}{4R_{p}h}\right]S,$$

In this study, both methods were used to determine the Young’s modulus of the fiber mats.

### 2.7. Cell Culture Studies and Smooth Muscle Cell Differentiation on Fiber Mats

Mouse embryo multipotent mesenchymal progenitor cells (C3H/10T1/2 cells; ATCC; Manassas, VA, USA) and mesenchymal stem cells derived from induced pluripotent stem cells (iMSCs, kindly donated by Dr. Dale Laird, Western University, London, ON, Canada) were used in this study. The fiber mat scaffolds were cut with aluminum foil supports, and the edges were folded to secure the mat onto the foil, creating an effective area of approximately 1 cm^2^. The fixed fiber mats were placed in individual wells of a 24-well plate and sterilized with 70% ethanol for at least 30 min, followed by three washes with HBSS before cell seeding.

For the 10T1/2 cells, they were directly seeded onto the surface of the fiber mat at a density of 20,000 cells/cm^2^ and cultured in high glucose DMEM supplemented with 5% fetal bovine serum and 1% penicillin/streptomycin.

Prior to iMSC seeding, the sterilized fiber mats were coated with 0.1% gelatin and incubated at 37 °C for 1 h. The iMSCs were then seeded onto the gelatin-coated fiber mats at a density of 20,000 cells/cm^2^ and cultured in mesenchymal stem cell expansion media (MSCEM, Cedarlane Labs, Burlington, ON, Canada; HMSC.E. MEDIA-450) supplemented with 10% fetal bovine serum, 1% L-glutamine, and 1% penicillin/streptomycin (all from Fisher Scientific, Whitby, ON, Canada). The cells were incubated at 37 °C in a humidified incubator containing 5% CO_2_.

To induce the differentiation of the iMSCs towards smooth muscle cell lineage, L-ascorbic acid (L-AA, Sigma-Aldrich Canada Co., Oakville, ON, Canada) and transforming growth factor-beta 1 (TGF-β1, R&D Systems, Minneapolis, MN, USA) were used, following a previously reported protocol [23]. After seeding the iMSCs on the fiber mat, the cells were pre-cultured on the fiber mats for 3 days until reaching approximately 70% confluence. The culture media were then changed to a differentiation medium comprising high glucose DMEM (Dulbecco’s Modified Eagle Medium) supplemented with 1% FBS, 1% penicillin/streptomycin, 82.5 µg/mL L-AA, and 2 ng/mL TGF-β1.

### 2.8. Cell Viability on Fibrous Scaffolds

The cell viability of the scaffolds was assessed using both live/dead staining and the MTT assay. After seeding the 10T1/2 cells onto the scaffolds for 7 days, the live/dead assay was conducted using a Live/Dead Cell Imaging Kit (488/570, Molecular Probes, Eugene, OR, USA; Life Technologies Corp., Carlsbad, CA, USA) following the manufacturer’s instructions. The cells were incubated with the working solution for 15 min at room temperature and, subsequently, the samples were examined using a 20× objective epifluorescence on Leica DMi8 (Leica Microsystems, Wetzlar, Germany) microscope equipped Leica Application Suite X (LAS X) software.

To evaluate the metabolic activity of viable cells, colorimetric assays were conducted using 3-(4,5-dimethylthiazol-2-yl)-2,5-diphenyltetrazolium bromide (MTT) (Invitrogen, Burlington, ON, Canada). After seeding the cells onto the scaffold for 2 days, 4 days, and 7 days, the scaffolds were transferred to a new 24-well plate to eliminate the influence of cells growing on the plate. Then, 100 μL of fresh medium and 10 μL of a 5 mg/mL MTT solution were added to each well. The samples were incubated at 37 °C for 4 h, during which time the yellow MTT salt was converted to a purple insoluble formazan salt by the dehydrogenase activity of intact mitochondria in metabolically active cells. Subsequently, 100 μL of sodium dodecyl sulfate (Invitrogen, Burlington, ON, Canada) was added and incubated for an additional 4 h to dissolve the formazan salt. Finally, the formazan salt was quantified at 570 nm (maximum absorbance) using a BioTek EL307C multi-plate reader (BioTek Instruments, Winooski, VT, USA).

### 2.9. Immunofluorescence Microscopy

After 7 days of culturing or 7 days of differentiation, the MSCs were fixed at 4 °C with 4% paraformaldehyde for 15–20 min, permeabilized with 0.5% Triton X-100 in PBS for 10 min, and then blocked in 2% BSA with 22.52 mg/mL of glycine in PBS for 30 min. Then, the samples were immunostained for 1 h at room temperature for α-SMA (mouse, 1:100; Santa Cruz, CA, USA, sc-32251), SMTN (rabbit, 1:100; Santa Cruz, sc-166292), and elastin (mouse, 1:100; Santa Cruz, sc-166543). Alexa Fluor^®^ 555 goat anti-rabbit IgG or Alexa Fluor^®^ 488 goat anti-mouse IgG was used as the secondary antibody for fluorescent detection (1 h incubation, 1:100 dilution; all from Life Technologies, Burlington, ON, Canada) and F-actin was detected with Alexa Fluor^®^ 568 or Alexa Fluor^®^ 488 phalloidin (1 h incubation, 1:100 dilution; Invitrogen Canada, Burlington, ON, Canada). Lastly, cell nuclei were visualized with 4′,6-diamidino-2-phenylindole (DAPI, 300 nM in PBS, Life Technologies, Burlington, ON, Canada). Coverslips were mounted on microscope slides with PermaFluor™ Mounting Medium (Thermo Scientific™, Mississauga, ON, Canada) and sealed with clear nail enamel. Images were taken with a Leica DMi8 (Leica Microsystems, Wetzlar, Germany) equipped with a 20× objective and analyzed using Leica Application Suite X (LAS X) software.

### 2.10. Quantitative Real-Time qPCR

Total RNA was isolated using a TRIzol™ reagent (Life Technologies) according to the manufacturer’s instructions. A total of 1 μg of RNA was used to synthesize cDNA using the Promega™ Random Hexamer protocol (Fisher Scientific, Whitby, ON, Canada). Real-time quantitative PCR (RT-qPCR) was conducted in 10 μL reaction volumes, using a CFX96™ Real-Time System (C1000 Touch Thermal Cycler; Bio-Rad, Mississauga, ON, Canada). The qPCR system contained 1 μL of cDNA, 0.2 μL of primers, 5 μL of SsoAdvanced Universal SYBR^®^ Green Supermix (Bio-Rad), and 3.8 μL of RNase-free water. The primer sequences are listed in Table 1.

### 2.11. Statistical Analysis

The data are expressed as a mean and a standard deviation. A minimum of three independent experiments were performed, and statistical analysis was conducted using a one-way ANOVA combined with Student’s two-sided independent sample *t*-test. Statistical significance was considered when the *p*-value was less than 0.05.

## 3. Results and Discussion

### 3.1. Characterization

**Figure 1:**
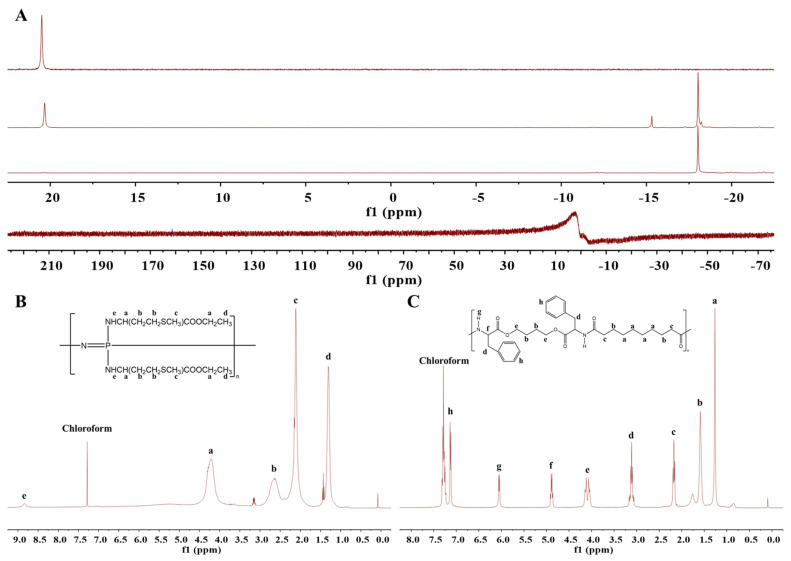
(**A**) ^31^P NMR of (1) HCCP, (2) crude PDCP from the flame-sealing method, and (3) purified PDCP, (4) PαAPz-M. (**B**) ^1^H NMR of PαAPz-M. (**C**) ^1^H NMR of PEA.

The ^31^P-NMR spectra of HCCP, linear PDCP, and PαAPz, and the ^1^H-NMR spectrum of PαAPz-M and PEA are shown in Figure 1A–C. Purified HCCP exhibited a singular peak at 21.09 ppm, whereas linear PDCP, following thermal ring-opening polymerization and purification, displayed a peak at −18.09 ppm. These characteristic peak positions concur with prior literature [32], thus confirming the successful synthesis of linear PDCP via the flame-sealing method. Upon the functionalization of PDCP with H-Met-OEt·HCl, the characteristic peak at −18.09 ppm vanished, and a new peak with diminished intensity emerged, which aligns with previous findings, thereby corroborating the success of the functionalization process [11].

In the ^1^H-NMR spectrum of PαAPz-M (Figure 1B), characteristic peaks associated with methionine ethyl ester were discerned between 0 and 5.0 ppm. The protons in -CH_2_-S-CH_3_ and ethyl carboxylate manifested peaks at approximately 4.3 and 2.1 ppm, respectively, indicating that the functionalization was successful and that the methionine ethyl ester remained unaltered during the functionalization process.

The ^1^H-NMR spectrum of PEA is presented in Figure 1C, and the characteristic peaks of PEA were found to agree with previous studies [33].

### 3.2. Scaffold Fabrication by Electrospinning

Electrospinning is one of the most frequently used methods in tissue engineering applications. Compared to other methods for scaffold formation (e.g., gas forming, solvent casting, and freeze-drying), electrospinning holds greater potential for large-scale industrial manufacturing of extracellular matrix (ECM)-like structures [34]. Furthermore, the technique may be combined with 3D printing to increase printing resolution [35,36]. However, compared to the other techniques, the electrospinning process has an increased complexity and imposes specific solubility and conductivity properties on each polymer–solvent combination that can be successfully used. Therefore, the potential electrospinnability of a new material can greatly increase its attractiveness in tissue engineering.

**Figure 2:**
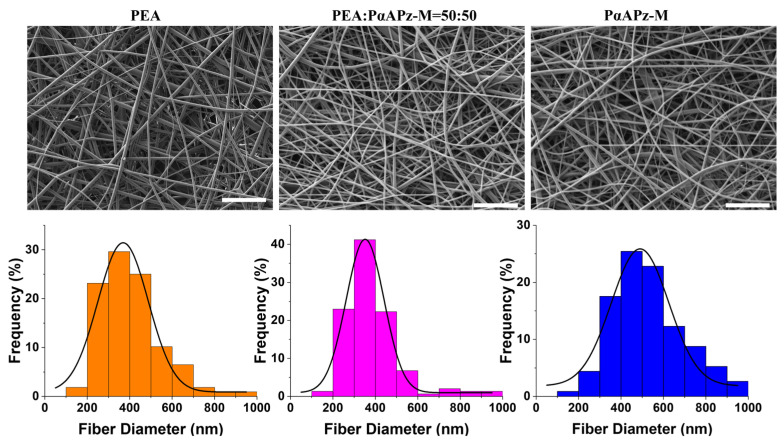
SEM images of electrospun PαAPz-M, PEA, and a mixture of PαAPz-M and PEA and the corresponding histogram showing the fiber diameter distribution. Scale bar = 10 μm.

To determine the conditions for producing bead-free electrospun fibers, screening experiments were conducted using various solvent combinations based on CHCl_3_ and THF at polymer concentrations ranging from 5% to 20% and a fixed flow rate and distance to the collector of 0.4 mL/h and 12 cm, respectively. The most optimal conditions for producing bead-free electrospun fibers of PαAPz-M were found to involve a mixed solvent of CHCl_3_:THF = 1:9 with a concentration between 10 and 15% and a fixed voltage of 15 kV. When the solution concentration was below 10%, electrospray occurred due to decreased viscosity; conversely, when the concentration exceeded 15%, the solution dried on the needle tip, resulting in blockage. To comparatively assess the degradation performance and cell adhesion of PαAPz-M with a previously studied material, we also electrospun PEA, a well-documented synthetic biomaterial utilizing amino acid as one of the constituent monomers. The electrospinning parameters for PEA were consistent with those previously reported [37]. In line with prior research showcasing the electrospinnability of PαAPz and PEA with chloroform [12,37,38], we employed chloroform (CHCl_3_) as the predominant solvent for electrospinning the blend. By trial and error, we determined that a CHCl_3_:DMSO solvent ratio of 3:1 with a distance of 12 cm, concentration of 15 wt%v, and voltage of 15 kV yielded optimal results, producing bead-free fibers. As illustrated in Figure 2, bead-free fibers with an approximate diameter of 400 nm were produced by pure PαAPz-M, PEA, and a blend of PαAPz-M and PEA.

### 3.3. Mechanical Properties of Fibers from AFM Measurements

Understanding fiber mat mechanical properties at the submicron scale, as probed by AFM, could be crucial for their application in tissue engineering. To this end, the utilization of AFM to measure the mechanical properties of decellularized ECM has been reported [39,40]. However, to our knowledge, its application in assessing the mechanical properties of porous synthetic fibrous scaffolds resembling ECMs has not received much attention.

**Figure 3:**
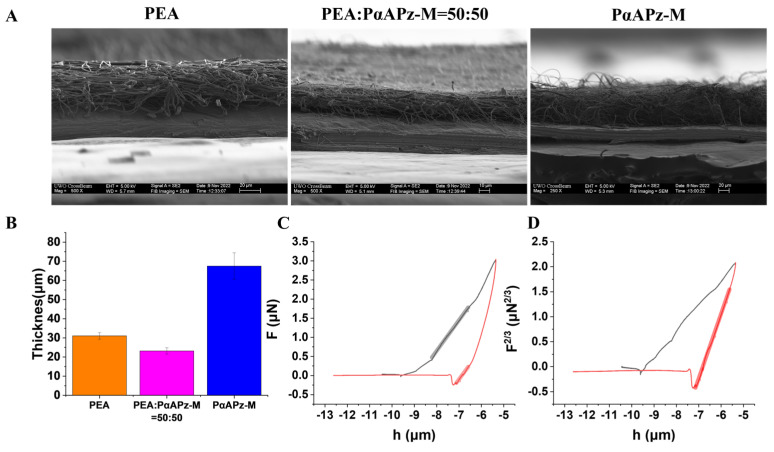
(**A**) SEM images of thickness of PαAPz-M, PEA, and mixture of PαAPz-M and PEA mats; (**B**) fiber mat thickness; (**C**) force curves during approach (black) and retraction (red) of AFM probe; (**D**) force curves of *F*^2/3^ vs. *h*. Area in bold is used for fitting and calculating Young’s modulus.

It is pertinent to mention that some studies have employed AFM to measure the mechanical properties of natural ECMs and porous membranes with structural similarities to our fiber mats [19,29]. Additionally, within the realm of polymer systems, both AFM and traditional mechanical tensile testing methods have yielded comparatively similar results [41,42]. These earlier investigations have laid a foundational understanding of the methods we employ to determine the Young’s modulus of our fiber mats. In this study, we employed the Hertz model to calculate the Young’s modulus of the fiber mats using two different approaches [28]. To ascertain the appropriate application of the Hertz model, we measured the thickness of the fiber mat employing scanning electron microscopy (SEM), as demonstrated in Figure 3A, and determined it to be between 30 and 70 μm (Figure 3B). When compared to the probe (diameter 7 nm), it was estimated that the material’s thickness was considerably greater, rendering the Hertz model applicable to this study.

Equations (2) and (5), derived from the Hertz model, indicate that when the contact area remains constant, *F*^2/3^ and h exhibit a linear relationship, while the *F*-*h* curve is non-linear. When the contact area varies with force, *F* and *h* display a linear relationship in the force curve [28]. Figure 3C illustrates the force–distance curve obtained after using AFM to examine the fiber mat. The approach force curve (black) is a segmented broken line, while the retraction force curve (red) is smooth. This suggests that when the fiber mat is compressed, the probe continuously penetrates the fiber, exhibiting a straight line. Simultaneously, the slope abruptly changes as the force increases due to its interaction and contact with other fibers, resulting in a broken line. In contrast, the retraction force curve maintains a constant contact area during retraction due to the material’s adhesion to the probe, resulting in a smooth curve. Figure 3D is derived from plotting *F*^2/3^ and *h*, demonstrating that the red retraction force curve is linear in a larger interval of deformation, as mentioned above.

In a retraction force curve, a slope near zero force is considered a reliable representation of the material’s Young’s modulus [31]. The bold lines in Figure 3C,D illustrate that F^2/3^-h represents the average mechanical properties observed in the retraction curve, while the F vs. h fitting captures the local mechanical properties experienced when the surface of the fiber mat undergoes compression or stretching on a submicron scale. The high slope observed at the end of the retraction curve is commonly attributed to the viscous resistance exhibited by the material and the tip. Thus, the Young’s modulus calculated using the F^2/3^-h model could be significantly higher than that of the F-h model. Despite the excellent fit achieved by the F^2/3^-h model, the local mechanical properties shown by F-h fitting offer a more direct reflection of the cellular microenvironment.

Consequently, we analyzed both the *F*^2/3^ vs. *h* and *F* vs. *h* slopes and compared the Young’s modulus calculated using the two slopes (as shown in Table 2). PαAPz-M displayed a significantly lower elastic modulus (0.4–0.8 GPa) than PEA (1.7 GPa), previously reported PCL scaffolds (1–4 GPa) [43], and polystyrene scaffolds (1.7 GPa) [44]. However, it still surpassed the elastic modulus of decellularized ECMs (pig heart 28–40 kPa and mouse lung parenchyma 25–30 kPa [19]). The reduced stiffness of PαAPz-M brings it closer to the mechanical properties of natural ECMs.

### 3.4. Fiber Mat Degradation and Morphology

The biodegradability of tissue engineering materials is crucial for the success of regenerative therapies. An ideal degradation rate must balance between supporting tissue regeneration, keeping the scaffold porosity, and avoiding adverse reactions [45,46]. In the early stages of cell culture in tissue engineering, the surface refresh rates and porosity changes of materials during degradation are of utmost importance. Therefore, we utilized SEM to evaluate the degradation behavior of the fiber mats during a 21-day incubation experiment in PBS at 37 °C.

**Figure 4:**
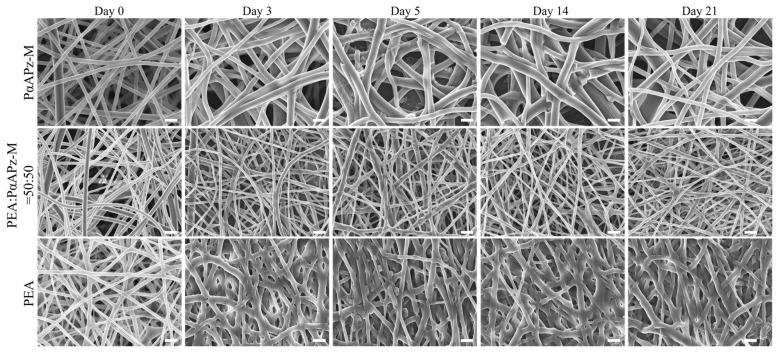
SEM images of morphology changes in electrospun mats fabricated from PαAPz-M, PαAPz-M/PEA blend, and PEA during 21 days of in vitro degradation. Scale bar = 2 μm.

As illustrated in Figure 4, PαAPz-M’s fiber diameter significantly increased by day 3, suggesting water penetration and swelling. For blended fiber mats with 50% PEA added, the fiber diameter remained relatively unchanged during degradation, although some slight fusion was observed. In contrast, the fiber mat composed solely of PEA fused the most during the 21-day culture period. Overall, the PαAPz-M and 50% blend maintained higher-porosity structures during the in vitro degradation.

The degradation performance of solid polymer surfaces is influenced by factors such as the hydrolysis rate of polymer bonds, water diffusivity, solubility, and material size [47,48,49]. Previous reports have raised concerns regarding the effect of the polyphosphazene side group on hydrolysis rate and cellular adhesion [11]. However, the degradation behavior of PαAPz-M is primarily characterized by swelling and bulk erosion, which may be attributed to the methionine hydrophilicity. This degradation pattern allows for a high degradation rate while maintaining a low surface refresh rate and preserving scaffold porosity. In contrast, the PEA scaffold exhibits more fusion, likely due to its higher solubility, which noticeably affects surface porosity during degradation. The 50% blend appears to strike a balance between solubility and water diffusivity, thereby maintaining favorable scaffold porosity throughout the 21-day degradation experiment.

### 3.5. Cell Viability and Adhesion on Fiber Mats

The ideal material for vascular tissue engineering should support vascular cells and promote good cell adhesion, cell viability, and differentiation. The viability, growth, and spreading of mesenchymal multipotent 10T1/2 cells on composite fibrous scaffolds were evaluated by MTT and live/dead assays.

**Figure 5:**
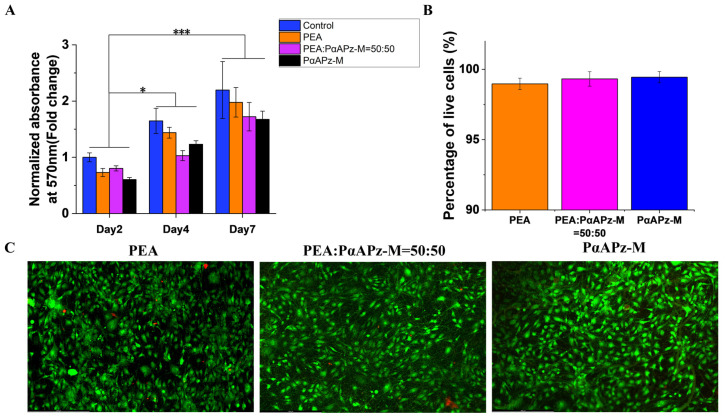
Cell viability of 10T1/2 cells on the fibrous scaffolds. (**A**) MTT assay on days 2, 4, and 7 of culturing; (**B**) percentage of living cells on the fibrous scaffolds; (**C**) live/dead assay of 10T1/2 cells on fiber mats. * *p* < 0.05; *** *p* < 0.001.

Figure 5A displays the cell viability of 10T1/2 cells on fibrous scaffolds on day 2, day 4, and day 7. The results showed that cell viability significantly increased over time, indicating an improvement in cell metabolism. Notably, no significant difference in cell viability among the three scaffolds was observed during the seven-day culture, suggesting that the degradation rate of PαAPz-M did not influence cell adhesion and proliferation. Although the viability of the cells cultured on the fiber mat appeared lower than that on the culture disk surface on day 2, the viability of the cells on the two surfaces was comparable after seven days of culture. These findings indicate that the fibrous scaffolds effectively support the growth and viability of 10T1/2 cells over time. In addition, the live/dead assay results (Figure 5B,C) demonstrated that after seven days of culture, 10T1/2 cells populated the fiber mat surface with only a small number of dead cells appearing on any of the three materials. This result confirms that both PαAPz-M and PEA materials exhibit good cell adhesion and viability and, therefore, have potential as biomaterials for vascular tissue engineering applications. Taken together, the results of this study suggest that PαAPz-M and PEA materials exhibit similar performance in terms of cell adhesion and cell viability and that both show promise as biomaterials for further research in artificial vascular tissue engineering.

### 3.6. Evaluation of MSC Adhesion and Smooth Muscle Cell Differentiation

**Figure 6:**
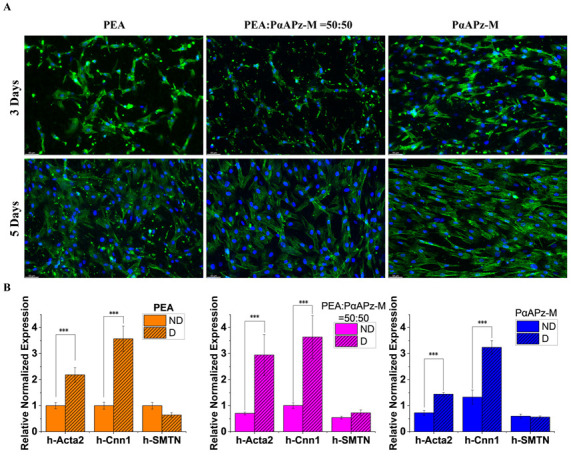
(**A**) Fluorescence images of MSC on PEA, PEA/PαAPz-M blend, and PαAPz-M fibrous mats after 3 days and 5 days of culture; F-actin is labeled by phalloidin in green and cell nuclei are labeled by DAPI in blue. (**B**) MSC mRNA expression of *hActa2*, *hCnn1*, and *hSMTN* was analyzed using RT-qPCR after cell treatment with L-AA and TGF-β1 for 7 days. ND stands for regular growth condition of iMSCs, and D stands for inducible condition of differentiation by exchanging culture medium to DMEM with 1% FBS, L-AA and TGF-β1. *** *p* < 0.001.

Building on our observations of 10T1/2 cells, we next investigated the effects of the increased degradation rate and associated surface changes on the adhesion, proliferation, and differentiation of MSCs on the fiber mats. Upon staining the MSCs after three and five days of cultivation, their morphology was examined. The data pointed to a robust adhesion of MSCs on all three material surfaces. A comparison of day 3 and day 5 images (Figure 6) revealed healthy cell spread on the material surfaces and a significant increase in cell nuclei, suggesting an active proliferation. These results imply that the surface changes occurring during the degradation of the PαAPz-M fiber mat do not significantly impede cell adhesion and proliferation. However, the low porosity caused by the degradation of the PEA fiber mats can potentially impact the infiltration of cells.

To investigate the differentiation capacity of stem cells, we employed L-ascorbic acid (L-AA) and TGF-β1 to instigate their differentiation into smooth muscle cells on the mats. Following seven days of differentiation culture, the expression of smooth muscle marker genes, such as h-Acta2, h-cnn1, and SMTN, was examined by q-PCR. A regular growth condition (ND) on the fiber mats served as a control. The findings indicated a notable upregulation of Acta2 and cnn1 (*p* < 0.05) after seven days of differentiation culture, although no substantial increase in SMTN expression was noted on any of the three materials (*p* > 0.05). Importantly, no significant differences were observed among the three materials. These findings further suggest that the degradation products of PαAPz-M and the presence of phosphate as a degradation product on the material surfaces did not adversely affect the differentiation of MSCs into smooth muscle cells (*p* > 0.05).

**Figure 7:**
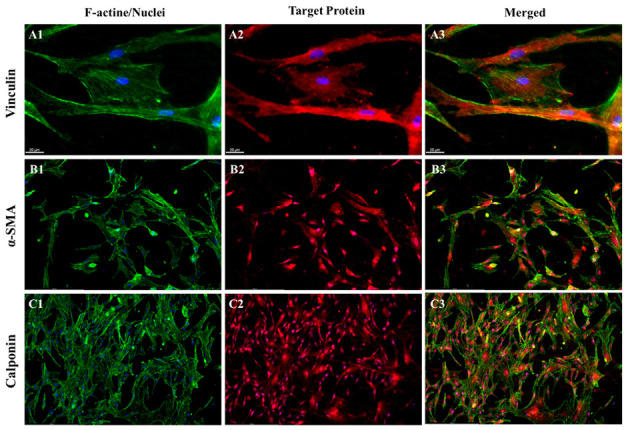
Immunofluorescence analysis of iMSC differentiation on PαAPz-M fibrous mats with the addition of L-AA and TGF-β1 in DC culture medium after 7 days of pre-differentiation; vinculin (**A1**–**A3**), α-SMA (**B1**–**B3**), and MHC (**C1**–**C3**) are labeled in red. F-actin is labeled by phalloidin in green and nuclei are labeled by DAPI in blue.

Despite the optimal performance observed in degradation experiments for the 50% mixed fiber mat, no significant differences were observed among the three materials in terms of gene expression. Therefore, for protein expression analysis, we specifically selected the PαAPz-M fiber mat, as it was exclusively reported in this study. After seven days of differentiation with L-AA and TGF-β1, the protein markers vinculin (red, Figure 7(A2)), α-SMA (red, Figure 7(B2)), and MHC (red, Figure 7(C2)) were detected. Vinculin, an essential player in cell adhesion to the ECM, was localized to adhesion plaques. Comparing F-actin and vinculin staining in Figure 7A, focal contacts were clearly visible. Figure 7B,C clearly demonstrates the robust expression of α-SMA and MHC within the cells. These findings further bolster the strong cell adhesion and SMC differentiation of MSCs on PαAPz-M at the protein expression level.

### 3.7. Analysis of ECM Expression

A key strategy in tissue engineering involves the use of biodegradable materials, with the anticipation that cells will generate their own extracellular matrix (ECM) during the material degradation process [50,51]. Because of this, we next examined the expression of collagen and elastin in MSCs on PαAPz-M fiber mats. For this series of experiments, we employed the same culture medium used in the differentiation culture. L-AA, present in this differentiation culture medium, is widely recognized to promote ECM expression [52,53]. In native blood vessels, collagen imparts tensile strength, while elastin provides elasticity, both crucial for the functional integrity of the vessel walls [54]. Despite various methods yielding tissue-engineered grafts with tubular structures, many reports have noted a deficiency in elastin expression [55,56]. Thus, the challenge of ensuring adequate elastin expression persists in vascular tissue engineering.

**Figure 8:**
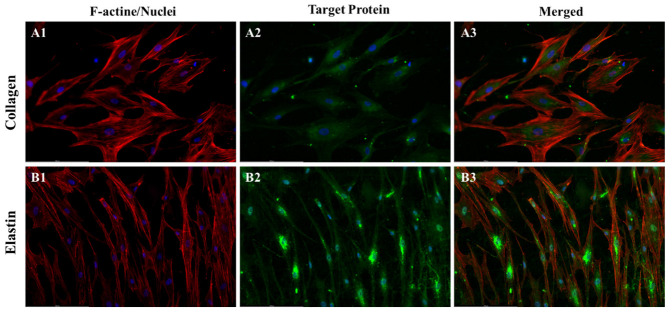
Immunofluorescence analysis of ECM expression by iMSCs on PαAPz-M fibrous mats. (**A1**,**B1**) show F-actin (red) and nuclei (blue); (**A2**) displays collagen (green); (**B2**) highlights elastin (green); (**A3**,**B3**) are merged images.

In a prior study, we observed that 10T1/2 cells expressed elastin on polyurethane and PEA [12,57]. Nonetheless, in the current study, as shown in Figure 8, the evident expression of elastin in h-MSCs, a viable candidate for vascular substitutes, compared to 10T1/2 model cells, was a particularly exciting observation. The expression of collagen and elastin provides strong evidence of ECM formation during the degradation of PαAPz-M. This outcome further bolsters the potential of PαAPz-M in vascular tissue engineering applications.

## 4. Conclusions

Our study concludes that PαAPz-M shows promising potential as a biomaterial in vascular tissue engineering. It exhibits mechanical properties closer to those of the natural extracellular matrix (ECM) compared to other tested materials. While PαAPz-M fiber mats can maintain high porosity during degradation, blending them with a hydrophobic material can help reduce swelling. Moreover, these scaffolds support the viability, proliferation, and smooth muscle cell differentiation of mesenchymal stem cells (MSCs), key elements for successful tissue regeneration. Furthermore, MSCs on PαAPz-M fiber mats show collagen and elastin expression, suggesting possible ECM formation during material degradation. These results make PαAPz-M a promising candidate for future research and application in vascular tissue engineering. However, there are some limitations to our study that warrant further studies. First, for ECM deposition, our culture time of 14 days is relatively short, as matrix deposition and assembly often require extended maturation times. Second, mechanical forces (e.g., fluid shear stress) that are known to influence cell behavior and matrix deposition are missing from our study since we used a circular mat instead of a tubular scaffold. These research avenues hold significant promise for PαAPz-M in vascular tissue engineering.

## Figures and Tables

**Table 1:** The primer sequences used in qPCR assay.

Gene	Forward Sequence (5′-3′)	Reverse Sequence (5′-3′)
h-Acta2	-CAA GTG ATC ACC ATC GGA AAT G	-GAC TCC ATC CCG ATG AAG GA
h-Cnn1	-TGA AGC CCC ACG ACA TTT TT	-GGG TGG ACT GCA CCT GTG TA
h-SMTN	-CAG GAC AAC AAG GAG AAC TGG	-CAG TCA ATT CCT CCA CAT CGT
h-18s	-GCG GTT CTA TTT TGT TGG TTT	-CTC CGA CTT TCG TTC TTG ATT

**Table 2:** Young’s modulus calculated from *F*-*h* fit and *F*^2/3^-*h* fit.

	Young’s Modulus (GPa)	
	From *F*-*h*	From *F*^2/3^-*h*
0PEA 100 PαAPz-M	0.440 ± 0.086	0.884 ± 0.076
50PEA 50 PαAPz-M	1.340 ± 0.141	4.907 ± 0.338
100PEA 0 PαAPz-M	1.749 ± 0.034	11.436 ± 0.816

## Data Availability

The data presented in this study are available in this article.
